# Electroacupuncture Confers Antinociceptive Effects via Inhibition of Glutamate Transporter Downregulation in Complete Freund's Adjuvant-Injected Rats

**DOI:** 10.1155/2012/643973

**Published:** 2012-08-23

**Authors:** Ha-Neui Kim, Yu-Ri Kim, Ji-Yeon Jang, Hwa-Kyoung Shin, Byung-Tae Choi

**Affiliations:** Division of Meridian and Structural Medicine, School of Korean Medicine, Pusan National University, Gyeongnam 626-870, Republic of Korea

## Abstract

When we evaluated changes of glial fibrillary acidic protein (GFAP) and two glutamate transporter (GTs) by immunohistochemistry, expression of GFAP showed a significant increase in complete Freund's adjuvant (CFA)-injected rats; however, this expression was strongly inhibited by electroacupuncture (EA) stimulation. Robust downregulation of glutamate-aspartate transporter (GLAST) and glutamate transporter-1 (GLT-1) was observed in CFA-injected rats; however, EA stimulation resulted in recovery of this expression. Double-labeling staining showed co-localization of a large proportion of GLAST or GLT-1 with GFAP. Using Western blot, we confirmed protein expression of two GTs, but no differences in the mRNA content of these GTs were observed. Because EA treatment resulted in strong inhibition of CFA-induced proteasome activities, we examined the question of whether thermal sensitivities and GTs expression could be regulated by proteasome inhibitor MG132. CFA-injected rats co-treated with EA and MG132 showed a significantly longer thermal sensitivity, compared with CFA-injected rats with or without MG132. Both EA and MG132 blocked CFA-induced GLAST and GLT-1 downregulation within the spinal cord. These results provide evidence for involvement of GLAST and GLT-1 in response to activation of spinal astrocytes in an EA antinociceptive effect. Antinociceptive effect of EA may be induced via proteasome-mediated regulation of spinal GTs.

## 1. Introduction

Glutamate is a main excitatory neurotransmitter in the spinal cord of mammals and, with its receptors, plays a key role in induction of central sensitization [1, 2]. An extremely high extracellular concentration of glutamate can act as a powerful neurotoxin; therefore, clearance of excess glutamate by glutamate transporters (GTs) is important under pathological conditions [3, 4].

GTs located in plasma membranes of neurons and glial cells are sodium-dependent excitatory amino acid transporters (EAATs) with a capacity for rapid uptake of synaptically released glutamate from the extracellular space and maintenance of glutamate homeostasis [5–7]. Total of five subtypes of EAATs have been cloned and characterized from animal and human tissues expressed mainly in neurons (excitatory amino acid carrier 1 (EAAC1/EAAT3)), EAAT4 and 5, and in glial cells [glutamate/aspartate transporter (GLAST/EAAT1) and glutamate transporter-1 (GLT-1/EAAT2)] [5, 8, 9].

Accumulating evidence has indicated another form of pain modulation arisen from nonneuronal glial cells [4, 10]. Under peripheral inflammation or injury, glial cells such as microglia and astrocytes have an important role in mediation of development and maintenance of central sensitization [11–14]. In particular, astrocytes, the most dominant cells in the central nervous system, play an essential role in pain modulation through rapid removal of glutamate from the synaptic cleft by GTs [2, 8, 15]. Inhibition of spinal GTs causes elevation in concentrations of spinal extracellular glutamate and results in spontaneous thermal and mechanical hypersensitivity [16, 17]. Dysfunction of astroglial GTs results in marked changes in spinal processing of nociceptive inputs [8, 18]; thus, functional enhancement of these transporters may be a suitable therapeutic target for management of pathological pain. 

Electroacupuncture (EA) has been widely used in the clinical setting for relief of acute and chronic pain with alterations of peripheral electrical stimulation rather than hand manipulation [19]. However, little is known about the relationship between the antinociceptive effect of EA and spinal GTs and their regulating factors following peripheral inflammation. 

We hypothesized that maintenance of spinal GTs expression and function is a key mechanism underlying EA analgesia in pain models. The complete Freund's adjuvant (CFA) has been utilized to induce rat inflammatory pain that displays many of the pathological features such as long-lasting pain [20–22]. In the present study, we examined the question of whether the antinociceptive effects of EA can antagonize activation of spinal astrocytes and downregulation of spinal GTs under conditions of CFA-induced inflammatory pain.

## 2. Materials and Methods

### 2.1. Animals and Injection of CFA

Male Sprague-Dawley rats with an average weight of 180 g were obtained from Dooyeoh Biotech (Seoul, Republic of Korea). Rats were housed at 22°C under alternating 12 h cycles of dark and light, fed a commercial diet, and allowed tap water ad libitum starting one week before the study and continuing throughout the study. Under light isoflurane anesthesia, rats received subcutaneous injection with 100 *μ*L of CFA (Sigma, St. Louis, MO, USA) into the plantar surface of the left hindpaw. CFA was injected only one time, following performance of a basal behavioral test. All experiments were approved by the Pusan National University Animal Care and Use Committee in accordance with the Council of the International Association for the Study of Pain of December 1982. Each group consisted of six rats and all treatments were administered under isoflurane (Choongwae, Seoul, Republic of Korea) anesthesia, using a model VIP 3000 calibrated vaporizer (Midmark, Orchard Park, OH, USA).

### 2.2. EA Stimulation

Under light gaseous anesthesia (1.0% isoflurane in air), two stainless-steel needles with a diameter of 0.2 mm were inserted to a depth of approximately 3 mm into each hind leg at acupoints corresponding to Zusanli (ST36) and Sanyinjiao (SP6) in men and were connected to an electrical stimulator (Pulsemaster Multi-Channel Stimulator SYS-A300, World Precision Instruments, Inc., Berlin, Germany). EA with 2 Hz stimulation of 1.0 mA was applied for 30 min once daily for 3 days, and gaseous anesthesia was discontinued three min prior to termination of EA treatment. For sham-EA control, acupuncture needles were inserted bilaterally at a point lateral to the aforementioned acupoints without electrical stimulation.

### 2.3. Intrathecal Injection

Lumbar catheterization was performed under 1% isoflurane anesthesia using a PE-10 intrathecal catheter inserted throughout the narrow space between the vertebra L5 and L6 levels in order to reach the lumbar enlargement of the spinal cord. Two days after surgery, only rats without overt signs of spinal cord or root damage, such as paralysis or lameness, were used for experimentation. Proteasome inhibitor MG132 (5 nmol, Calbiochem, Darmstadt, Germany), which was dissolved in 0.4% DMSO, was injected intrathecally at a volume of 10 *μ*L via a catheter within 1 min. The catheter was then filled with 8 *μ*L of saline for flushing. For EA stimulation, MG132 was administered twice daily into the subarachnoid space of the spinal cord at 5 min prior and 12 h intervals afterward. Using an identical method, the vehicle control group for MG132 received injections of identical amounts of 0.4% DMSO. 

### 2.4. Measurements of Thermal Sensitivity

The plantar test (Ugo Basile 37370, Comerio, Italy) was performed for measurement of paw withdrawal latency (PWL) of thermal. Rats were placed in six separated cages (17 × 11.5 × 14 cm high) for a period of 30 min after EA treatment and nociceptive thresholds of the left hindpaw were assessed three times, with a five min interval between trials; mean values were taken as the PWL. Intensity of the infrared generator was adjusted in order to produce withdrawal latencies of approximately 8–10 sec (infrared intensity of 80) in rats. A cutoff period of 15 sec was used. On day one of the experiments, the basal noxious threshold was measured, followed by injection with CFA according to the regular sequence of groups. PWL was then monitored at 30 mins after EA stimulation with or without MG132 treatment. Animals that did not receive EA treatment were subjected to the same anesthesia as those that received EA treatment, followed by measurement of PWL. 

### 2.5. Immunohistochemistry

The L4-5 segments of the spinal cords were fixed in 4% paraformaldehyde and immersed in 30% sucrose for 48 h at 4°C for cryoprotection. Frozen serial sections 14 *μ*m in thickness were prepared by the six to eight sections from each spinal cord. Sections were preincubated in 1% hydrogen peroxide for 20 min (only for c-fos staining), washed in PBS, and transferred to blocking solution (CAS-block, Invitrogen-Molecular Probes, Camarillo, CA, USA) for 10 min at room temperature. Sections were then incubated with the primary anti-fos antibody (Santa Cruz Biotechnology, Santa Cruz, CA, USA; 1 : 500) in PBS and anti-EAAT1 (GLAST) and anti-EAAT2 (GLT-1) (Cell Signaling Technology, Danvers, MA, USA), anti-glial fibrillary acidic protein (GFAP, Millipore, Billerica, MA, USA) in antibody dilution buffer (1X PBS, 1% BSA, 0.3% Triton X-100) at 4°C. After washing with phosphate buffered saline (PBS) containing Tween-20 (PBST), the sections were incubated in corresponding secondary antibody biotinylated anti-rabbit IgG (Vector Laboratories, Burlingame, CA, USA), goat anti-rabbit IgG-TR (Santa Cruz Biotechnology), or anti-mouse IgG-fluorescein isothiocyanate (FITC) (Vector Laboratories) for 1-2 h at room temperature, followed by washing with PBST. For c-fos staining, sections were incubated with ABC solution (Vectastain ABC kit, Vector Laboratories) in PBS for one h, followed by development in diaminobenzidine (DAB) peroxidase substrate (Vector Laboratories) for 5–20 min, followed by dehydration. For fluorescence examination, slides were mounted in permanent mounting medium or mounting medium (Vector Laboratories). To quantify expression, images of dorsal horns were captured using an Axio Vision (Carl Zeiss, Oberkochen, Germany) for c-fos and Axio Vision LSM 510 (Carl Zeiss) for GFAP, GLAST, and GLT-1. In order to quantify laminar expression, the dorsal horn of the spinal cord was divided into 3 regions: the superficial laminae (laminae I and II), the nucleus proprius (laminae III and IV), and the neck of the dorsal horn (laminae V and VI). The number of c-fos-like immunostained neurons was counted and averaged per section. IMT i-Solution (IMT i-Solution Inc., Vancouver, BC, Canada) was used for automatic measurement of integrated optical density (IOD) of the dorsal horn. For controls, treatment with primary and secondary antibodies was omitted.

### 2.6. Western Blot

For Western blot analysis, the L4-5 segments of the spinal cords were obtained after each experiment. Spinal cords were washed in cold HEPES buffer and homogenized in nine volumes of lysis buffer. Equal amounts of proteins were then separated by 8–12% sodium dodecyl sulfate-polyacrylamide gel electrophoresis, followed by the transfer of resolved proteins to a nitrocellulose membrane (Whatman, Dassel, Germany), which was subsequently blocked with 5% nonfat milk in Tris-buffered saline containing 0.4% Tween-20. Membranes were incubated with primary antibodies, as used in immunohistochemical analysis, for 1-2 h at room temperature; the blots were then incubated with horseradish peroxidase-conjugated secondary antibody, followed by visualization of antibody-specific proteins using an enhanced chemiluminescence detection system, according to the recommended procedure (Pierce, Rockford, IL, USA). *β*-actin was used as a loading control for all experiments. Densitometric analysis was performed for quantification of immunoreactivity corresponding to the total and phosphorylated bands using an Image Quant LAS 4000 (Fujifilm, Tokyo, Japan).

### 2.7. Reverse Transcription Polymerase Chain Reaction (RT-PCR)

Total RNA was prepared from spinal cord L4-5 using TRIZOL Reagent (Invitrogen, Paisley, UK), according to the manufacturer's protocols. cDNA was synthesized using 2 *μ*g of total RNA and oligo_dT(18)_ primer with taq polymerase (Promega) in a 20 *μ*L total reaction volume. Reverse transcription was performed by incubating the mixture at 37°C for 45 min, and the reaction was terminated at 95°C for five min. The following primers were used: 5′-GAAAGATAAAATATGCAAAAAGCAAC-3′ (forward), and 5′-GTTGCTTTTTTGTCATATTTTATCTTTC-3′ (reverse) for GLAST; 5′-ATCAACCGAGGGTGTGCCAACAATAT-3′ (forward) and 5′-ATATTGTTGGCACCCTCGGTTGAT-3′ (reverse) for GLT-1; and 5′-ATGAGAAGGAGATC-ACTGC-3′ (forward) and 5′-CTGCGCAAGTTAGGTTTTGT-3′ (reverse) for *β*-actin. For PCR, the reaction mixture was subjected to 30 cycles of 30 s at 94°C, 30 s at 55°C, and 60 s at 72°C. PCR products were then electrophoresed on 1.5% agarose gels and stained with ethidium bromide. All data were normalized to *β*-actin primers, and quantification was performed as described for Western blot analysis.

### 2.8. Proteasome Activity Assay

For measurement of proteasome activity, a Proteasome-Glo Chymotrypsin-Like Cell-Based Assay was used as a luminescence assay (Promega Corporation, Madison, WI, USA). Briefly, tissues were washed with PBS followed with ice cold homogenized buffer (10 mM Tris (pH 7.5), 5 mM MgCl_2_, 0.5 mM DTT, 5 mM ATP), and pelleted by homogenization. For removal of tissue debris, centrifugation was performed at 10,000 g for 20 min at 4°C. The Bradford protocol was used for determination of protein concentration to a total of 10 *μ*g/50 *μ*L of protein of each sample. Samples were transferred to 96-well plates, followed by addition of a mixture of luciferin detection reagent and an appropriate volume of Proteasome-Glo buffer. Then, for the chymotrypsin-like assay, Suc-LLVY-Glo Substrate, Proteasome-Glo Reagent, and Proteasome-Glo Substrate were added to total 150 *μ*L per well. Cleavage activity was monitored continuously by detection of free 7-amido-4-methylcoumarin using a Synergy HT Multi-Detection Microplate Reader (Bio-Tek Instruments, Inc., Winooski, VT) at 380/460 nm and 37°C. As a control, Suc-LLVY-Glo Substrate was incubated with each agent in homogenized buffer without cell extracts, followed by measurements of proteasome activity.

### 2.9. Data Analysis

Data are expressed as the mean ± SEM. Multifactorial analysis of variance (ANOVA) using the Sigmastat statistical program Version 11.2 (Systat Software, San Jose, CA, USA) was performed for analysis of data. A two-way ANOVA post hoc test via Tukey's test was performed for behavioral analysis. Western blot and immunohistochemical analysis, RT-PCR, and proteasome activity assay were performed using a one-way ANOVA post hoc test via Tukey's test. A *P* < 0.05 was considered statistically significant.

## 3. Results

### 3.1. Behavioral Analysis of CFA-Induced Thermal Sensitivity

**Figure 1:**
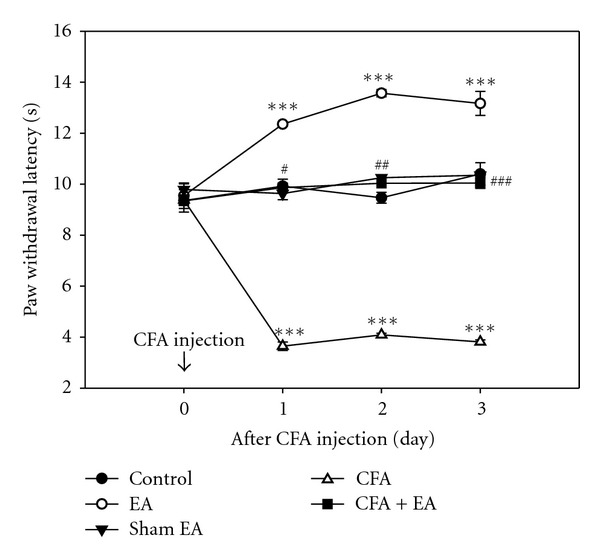
Effect of EA stimulation on thermal PWL. A significant antinociceptive effect was observed in CFA-injected rats with EA stimulation, compared with CFA-injected rats. Each point indicates the mean ± SEM (*n* = 6). ***P < 0.001 compared with control rats; ^#^ *P* < 0.05, ^##^ *P* < 0.01, and ^###^ *P* < 0.001 compared with CFA-injected rats.

To determine antinociceptive effects of EA, we measured PWL from a thermal stimulus. Thermal sensitivity of CFA-injected rats (CFA group) showed a significant decrease from days 1, 2, and 3, compared with control rats; however, CFA-injected rats with EA stimulation (CFA + EA group) showed a marked reversal of CFA-induced hyperalgesia. Only EA-stimulated rats (EA group) showed a significant increase of antinociception, compared with control rats; however, no effect was observed in sham-EA-stimulated rats (sham-EA group) (Figure 1). These results demonstrate a significant antinociceptive effect of EA on CFA-induced inflammatory pain.

### 3.2. Immunohistochemical Analysis for c-fos and GFAP Expression

**Figure 2:**
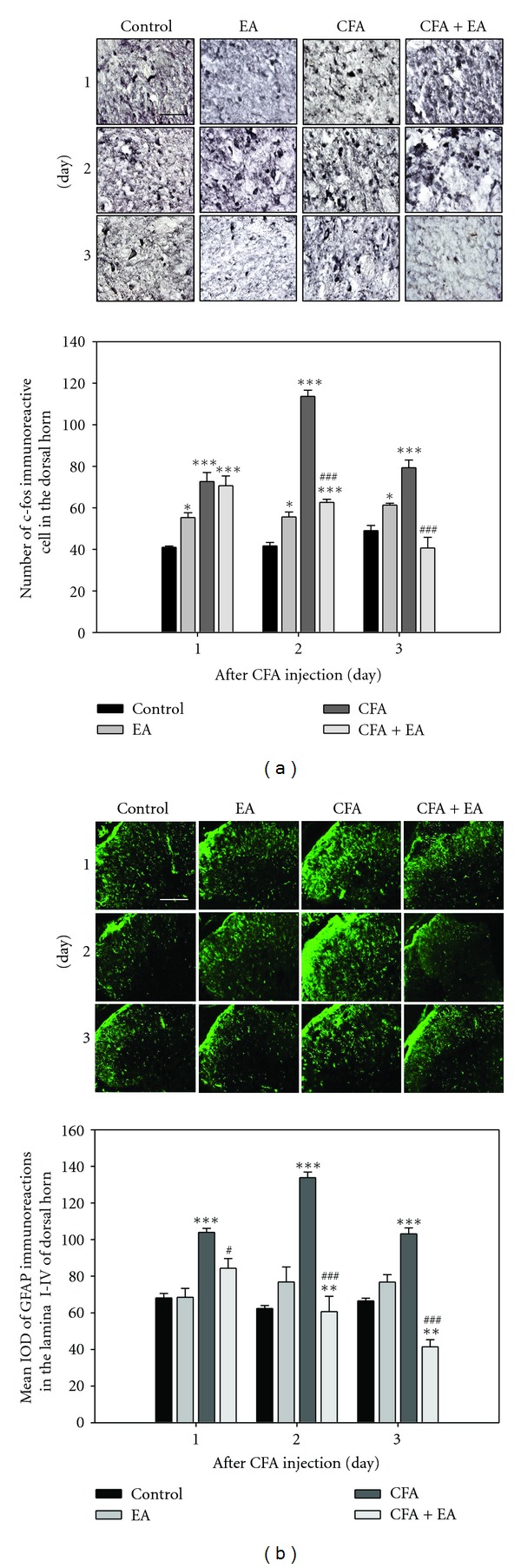
Immunohistochemical expression of c-fos (a) and GFAP expression (b) in the dorsal horn of the L4-5 segment (*n* = 5). Both c-fos and GFAP expressions showed a similar pattern, in that EA treatment resulted in strongly inhibited immunoreactions of both expressions elevated by CFA-injection. *P < 0.05 and ***P < 0.001 compared with control rats; ^###^ *P* < 0.001 compared with CFA-injected rats. Scale bar = 50 (a) and 200 *μ*m (b).

In order to explore the response to CFA-induced nociceptive stimuli, we examined the number of c-fos immunoreactive cells in the dorsal horn of the L4-5 segment. A significant increase in the number of c-fos immunoreactive cells was observed in CFA-injected rats, compared with control. However, a significant decrease in the number of c-fos immunoreactive cells was observed in CFA-injected rats with EA stimulation on days 2 and 3 after CFA-injection (Figure 2(a)). For examination of the time course of astrocyte activation induced by CFA, we used GFAP as a marker for activation. Strong expression of GFAP was observed in gray matter of the spinal cord, especially through laminae I–IV in the dorsal horn. Expression of GFAP showed a pattern similar to those of c-fos immunoreactions, in which EA stimulation resulted in attenuated CFA-induced astrocyte activation throughout the experiment (Figure 2(b)). These results suggest that EA treatment may induce an antinociceptive effect via downregulation of astrocyte activation in CFA-injected rats.

### 3.3. Immunohistochemical Analysis for GLAST and GLT-1 Expression

**Figure 3:**
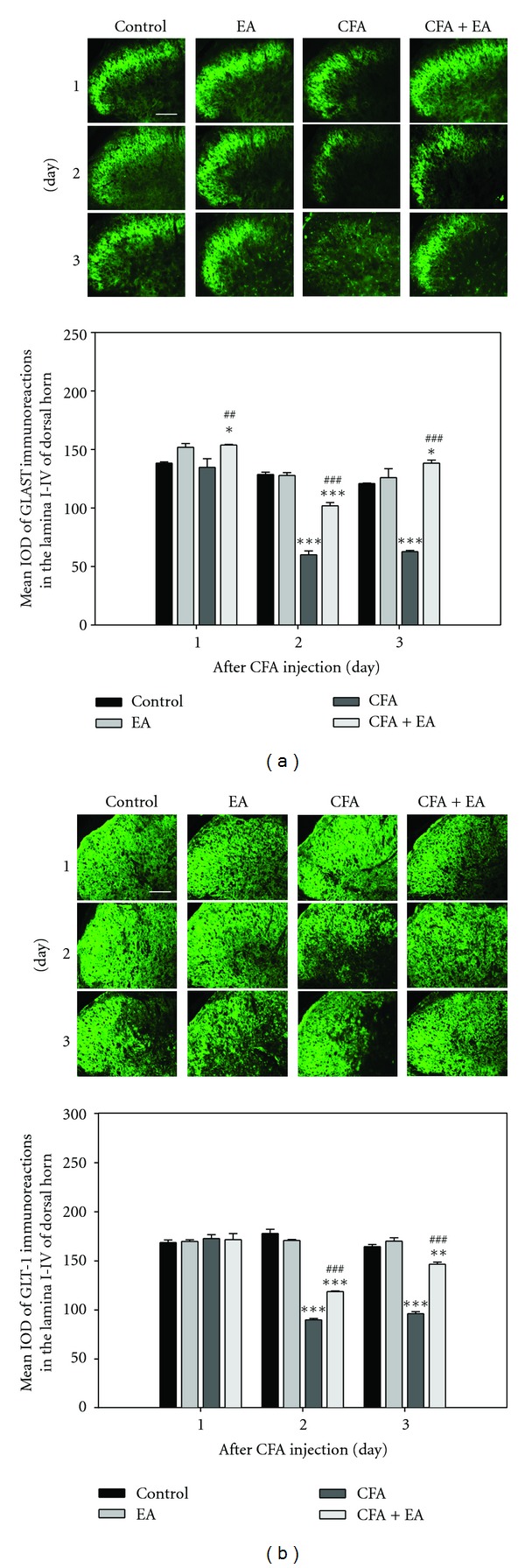
Immunohistochemical expression of GLAST (a) and GLT-1 (b) in the dorsal horn of the L4-5 segment (*n* = 5). Immunoreaction of both GLAST and GLT-1 showed a significant decrease in CFA-injected rats, compared with control; however, these reactions showed marked recovery by EA stimulation. *P < 0.05, **P < 0.01, and ***P < 0.001 compared with control rats; ^##^ *P* < 0.01 and ^###^ *P* < 0.001 compared with CFA-injected rats. Scale bar = 200 *μ*m.

**Figure 4:**
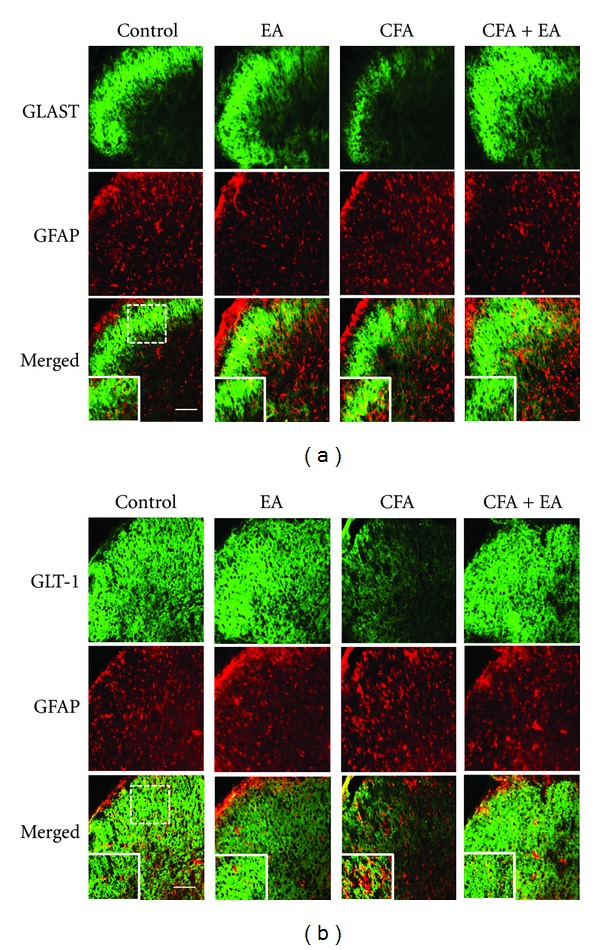
Double immunolabeling for GLAST (a) and GLT-1 (b) with astrocyte marker GFAP (*n* = 5). Double immunolabeling showed colocalization of both GTs with GFAP; these colocalizations were largely observed in CFA-injected rats. Small frame represents high-power merged image of GFAP and GLAST or GLT-1. Scale bar = 200 *μ*m.

To determine functional changes of spinal astrocytes, expression of GLAST and GLT-1, which play a critical role in glutamatergic regulation in the spinal cord, was evaluated. Expression of GLAST and GLT-1 in the spinal dorsal horn was detected mainly in gray matter of the spinal cord, particularly GLAST in laminae I–IV of the dorsal horn. Significant loss of expression of both GLAST and GLT-1 in CFA-injected rats was observed on days 2 and 3, compared with control. However, significant recovery of this expression was observed after EA stimulation (Figures 3(a) and 3(b)). To further explore localization of expression of two GTs, we employed a double immunolabeling analysis. Results of double immunofluorescence showed colocalization of a large proportion of both GLAST and GLT-1 with the astrocytic marker GFAP (Figures 4(a) and 4(b)), but not with the neuronal marker NeuN or the microglia marker OX-42 (data not shown). Marked colocalized expression was observed in CFA-injected rats. These results suggest that CFA injection can result in a decrease in total expression of GLAST and GLT-1 and that an antinociceptive effect of EA may be observed through maintenance of these GTs. In addition, results of the double-labeling study provide a clear evidence of regulated expression of GLAST and GLT-1 in astrocytes.

### 3.4. Western Blot and RT-PCR Analysis for GLAST and GLT-1

**Figure 5:**
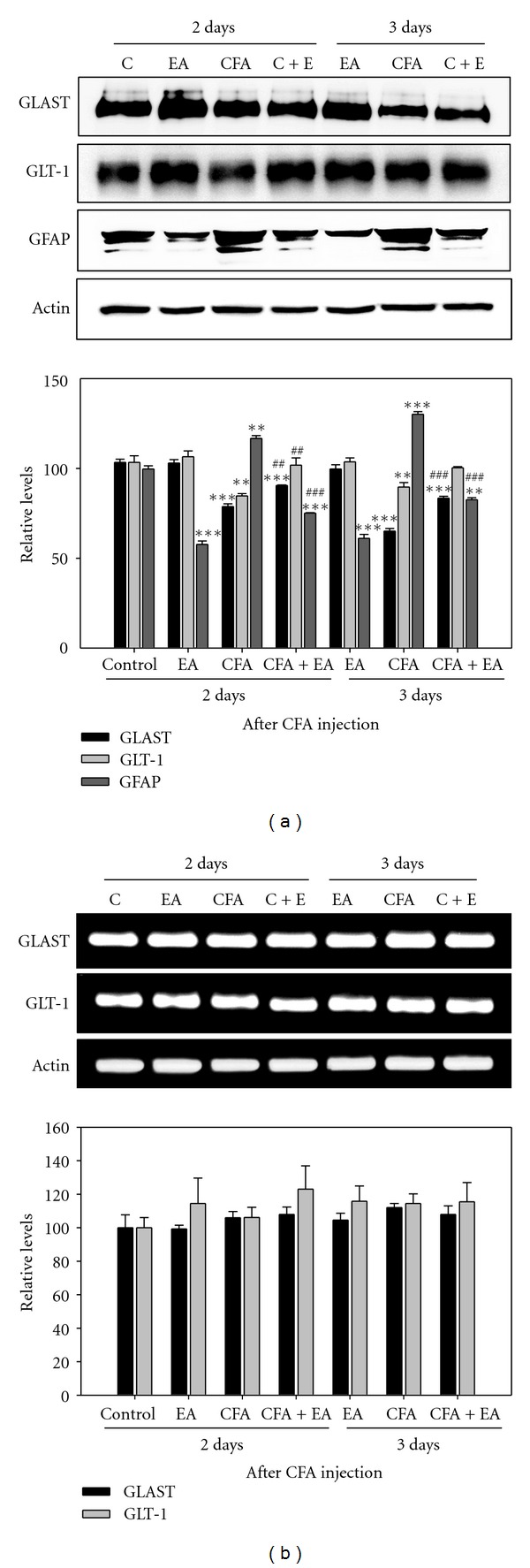
Western blot (a) and RT-PCR analysis (b) for GLAST and GLT-1 in the L4-5 segment of the spinal cord (*n* = 5). Expression of GLAST and GLT-1 protein caused by CFA showed significant recovery by EA stimulation, whereas that of GFAP was arrested. However, no differences in mRNA content of the GTs were observed among the four groups. The level of each protein is expressed as a percentage of the normal control. The panel represents a typical result from five independent experiments. **P < 0.01 and ***P < 0.001 compared with control rats; ^##^ *P* < 0.01 and ^###^ *P* < 0.001 compared with CFA-injected rats.

To explore the question of whether a beneficial effect of EA on CFA-injected rats occurs through protein or mRNA level of GTs, we performed Western blot and RT-PCR analysis for GLAST and GLT-1 on days 2 and 3 after CFA injection. For expression of GLAST and GLT-1 protein, significant decreases were observed in CFA-injected rats, compared with controls. However, these decreases of both GLAST and GLT-1 showed strong recovery with EA treatment on days 2 and 3, respectively. By contrast, expression of GFAP in CFA-injected rats was significantly higher than that in controls on both day 2 and day 3, and these increases were significantly attenuated by EA stimulation (Figure 5(a)). For expression of GLAST and GLT-1 mRNA, no differences were observed in the content of these GTs showing no downregulation at the mRNA level among the four groups (Figure 5(b)). These results indicate that CFA-induced inflammation may involve downregulation of GLAST and GLT-1 protein on the posttranscriptional level. And, EA may exert antinociceptive effects via inhibition of degradation of mainly these GT proteins.

### 3.5. Proteasome Activity Assay

**Figure 6:**
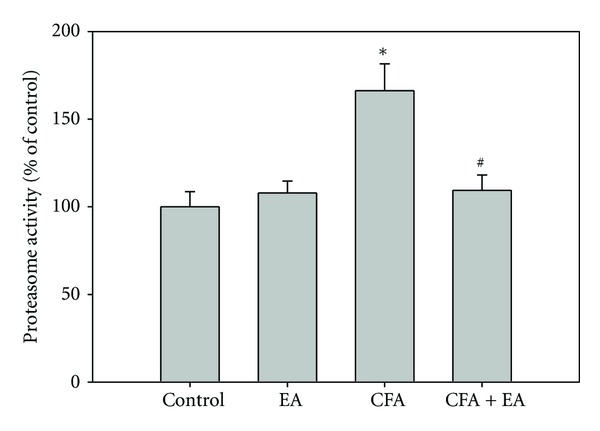
Effects of EA stimulation on proteasome activity among four groups on day 2 after CFA injection. Proteasome activity in CFA-injected rats was higher, compared to controls; however, CFA-injected rats with EA showed a result similar to that of controls. Data are expressed as the mean ± SEM (*n* = 5). *P < 0.05 compared with control rats; ^#^ *P* < 0.05 compared with CFA-injected rats.

We supposed that if downregulation of GLAST and GLT-1 occurred on the cellular protein level, not in mRNA, the ubiquitin-26S proteasome pathway may play an important role in posttranscriptional regulation of these proteins. We performed a proteasome activity assay between the four groups on day 2 after CFA injection. A significant increase in proteasome activity was observed in CFA-injected rats, compared to controls; however, that in CFA-injected rats with EA showed a response similar to that of controls (Figure 6). These results suggest that EA may exert antinociceptive effects via inhibition of proteasome-mediated degradation of GLAST and GLT-1.

### 3.6. Intrathecal Injection of Proteasome Inhibitor MG132

**Figure 7:**
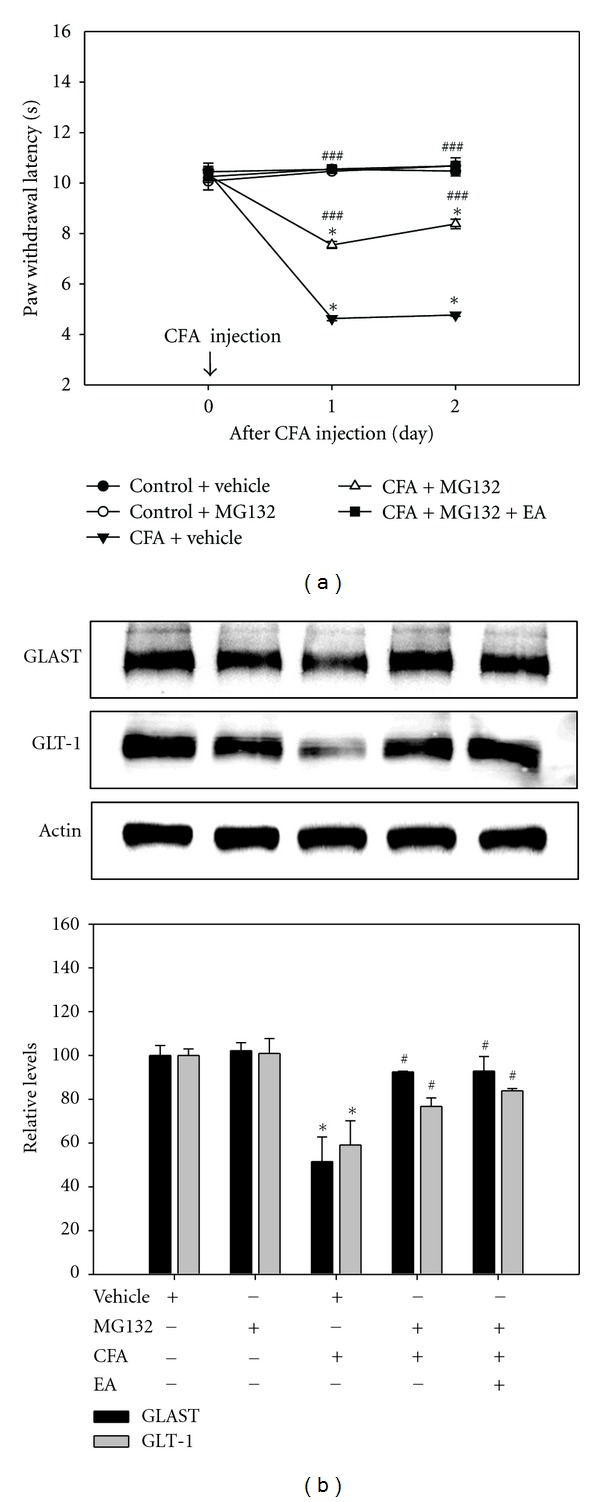
Effects of proteasome inhibitor MG132 on thermal PWL (a) and expression of GLAST and GLT-1 protein (b). CFA-injected rats treated with MG132 showed significant antinociception characterized by a higher PWL, compared with CFA-injected rats. In addition, CFA-injected rats cotreated with EA and MG132 showed a similar PWL, as with controls. Each point of PWL indicates the mean ± SEM (*n* = 6). Decreased expression of GLAST and GLT-1 caused by CFA was observed by both EA and MG132 treatments. *P < 0.05 compared with control rats with vehicle; ^#^ *P* < 0.05 compared with CFA-injected rats with vehicle.

To investigate the question of whether the proteasome inhibitor MG132 would modulate thermal sensitivity and regulate spinal GT proteins, we treated rats with MG132 and measured thermal PWL and expression of GLAST and GLT-1 protein. For thermal hyperalgesia, MG132-treated rats showed significant antinociception characterized by a higher PWL profile, compared with CFA-injected rats. CFA-injected rats treated with EA and MG132 showed a PWL similar to that of control rats (Figure 7(a)). Evaluation of expression of GLAST and GLT-1 protein on day 2 after CFA injection by Western blot showed recovery of decreased expression of GLAST and GLT-1 induced by CFA in both EA- and MG132-treated rats (Figure 7(b)). These results suggest that inhibition of proteasome-mediated GTs degradation by EA stimulation may be an important mechanism for prevention of CFA-induced nociception.

## 4. Discussion 

The EA stimulation is widely utilized in clinical treatment for relief of inflammation, neuropathic pain, and arthritis that were induced by CFA on experiment model [10, 21, 23]. In the central nervous system, EA induces an analgesic response mainly through diverse signal molecules including opioid peptides, glutamate, 5-hydroxytryptamine, and cholecystokinin octapeptide [19, 24]. Many studies investigated that EA stimulation markedly reduces inflammatory hyperalgesia by the inhibition of the release of glutamate and the expression of ERK, p38 in the spinal dorsal horn [10, 11, 24]. Induction of EA antinociception involves N-Methyl-D-aspartate receptor-related signaling and its regulation of calcium influx [25–27]. Among these signal molecules, GTs expression was involved with inflammatory pain in dorsal root ganglion and spinal cord [8, 13, 28]. Findings from additional studies have demonstrated that antinociceptive effects of EA may occur via functional enhancement of GTs in the spinal cord. However, EA-induced antinociception via spinal GTs has received relatively little attention. Therefore, the goal of the present study is to observe changes in the expression of spinal GTs resulting from EA stimulation in CFA-injected rats. 

Following peripheral inflammation by CFA, multiple changes in central sensitization occur, leading to thermal hyperalgesia by inflammation at the site of intraplantar injection [12]. First, we observed the inhibitory effects of EA stimulation on thermal PWL. Injection with CFA resulted in a condition of persistent thermal hyperalgesia throughout the experiments. Consistent with our previous results, application of EA stimulation for 30 mins once daily for three days resulted in significantly abolished CFA-induced hyperalgesia [29]. Following CFA treatment, observation of spinal c-fos-positive cells to confirm the response to nociceptive stimuli in the spinal cord showed a significant increase in the number of these cells in all layers of the spinal cord, particularly in laminae I-II and V-VI, consistent with previous results [21, 30]. However, this increase of c-fos was strongly inhibited by EA stimulation, demonstrating that EA stimulation results in a significant antinociceptive effect in CFA-injected rats. 

Findings from accumulating studies have demonstrated activation of both spinal microglia and astrocytes in diverse models of pathological pain. Early activation of spinal microglia and subsequent long-term activation of astrocytes contribute to induction and maintenance of persistent chronic pain [8, 10, 31]. Damage to peripheral tissues induces activation of astrocytes, and an altered state, resulting in upregulation of GFAP expression with hypertrophic morphology [8, 10].

Astrocytic activation may contribute to development and maintenance of hyperalgesia-like behaviors [28, 32]. We focused on activation of astrocytes using GFAP, which may be targeted for reduction of the persistent pain state. Significant upregulation of astrocytic GFAP expression in the spinal cord was observed throughout the experiment, especially on day 2 after CFA injection. However, this expression was strongly inhibited by EA stimulation. Astrocyte activation is known to be accompanied by downregulation of spinal GTs associated with development of pathological pain [8, 33, 34]. If activation of astrocytes is controlled effectively by EA, expression of GLT-1 and GLAST exclusively in astrocytes may play key roles in the prevention of pathological pain. We further elucidate changes of GLAST and GLT-1 in the dorsal horn. 

Wide expression of GLAST and GLT-1 was observed in gray matter of the spinal cord; in particular, GLAST is concentrated in laminae I–IV of the dorsal horn, an area important for sensory input and pain processing, consistent with findings from previous studies [18]. On the first day after CFA injection, no differences were observed among groups; however, expression of both GLAST and GLT-1 in CFA-injected rats showed a marked decrease during the next two days, compared with control. However, in the present study, EA stimulation resulted in significant blockade of CFA-induced downregulation of these GTs. Our results from double staining showed colocalization of both GLAST and GLT-1 with the astrocytic marker but not with the neuronal marker or the microglia marker. This finding is consistent with that of a previous report showing that under normal conditions in the spinal dorsal horn, these GTs originate primarily in astrocytes [4].

Spinal GTs prevent occurrence of glutamate overexcitation under a variety of types of pathological pain [8, 18]. Downregulation of GTs protein would result in elevation of extracellular glutamate levels at the synapse and is associated with allodynia and hyperalgesia [10, 34]. In addition, pharmacological inhibition of astrocytic activation by propentofylline leads to attenuated pathological pain and induces expression of GLT-1 and GLAST in the spinal cord [35, 36]. In addition, amitriptyline, used as a drug for treatment of neuropathic pain, has been reported to reverse downregulation of GLT-1 and GLAST in rats with nerve injury [37, 38]. When we consider upregulation or functional enhancement of these transporters for prevention of pathological pain, EA may play an important role in CFA-induced pain via upregulation of spinal GLAST and GLT-1.

Expression of GTs is regulated at the transcriptional, translational, and posttranslational levels [39]. We next investigated the question of whether EA stimulation could result in modulation of GTs gene transcription or protein expression in CFA-induced pain. Our results from RT-PCR and Western blot analysis suggested that EA induced effective recovery of CFA-induced decreased protein level; however, no differences in GTs mRNA content were observed. These results suggest that EA stimulation is not a direct result of GTs synthesis but rather is mediated by upregulation of GTs expression at the posttranscriptional level.

The ubiquitin-proteasome system (UPS) is a major nonlysosomal proteolytic pathway of selective degradation of cellular proteins and an important mechanism of posttranslational regulation [26]. Inhibition of ubiquitin-proteasome-mediated GTs degradation is an important mechanism for prevention of glutamate overexcitation under conditions of neurogenic and inflammatory pain [20, 40–42]. Regulation of both GLAST and GLT-1 expression by EA treatment could be caused by the UPS system. We attempted to determine whether activities of spinal proteasome would be modulated by EA in CFA-injected rats. Significantly increased proteasome activity was observed in CFA-injected rats; however, EA stimulation resulted in arrest of its activity, indicating proteasome involvement in downregulation of GTs.

We supposed that EA-induced antinociception in CFA-injected rats can be regulated by ubiquitin-proteasome-mediated GTs degradation. To confirm involvement of proteasome in development of thermal sensitivity and downregulation of GTs, we administered intrathecal injection of proteasome inhibitor MG132 and performed behavioral analysis and Western blot for GLAST and GLT-1. Findings from previous studies have demonstrated that proteasome inhibitor MG132 can prevent glutamate overexcitation in a chronic pain correlate with alterations in behavioral responses [20, 23, 40, 43]. These findings indicated that proteasome-mediated GTs degradation is required for maintenance of chronic pain.

MG132 was effective in prevention of CFA-induced thermal hypersensitivity in the hindpaw, suggesting a possible role of UPS in this process. In addition, cotreatment of MG132 and EA stimulation resulted in markedly increased thermal sensitivity, similar to control. In addition, we found that intrathecal administration of MG132 prevented downregulation of spinal GTs, as with EA. The present results demonstrate that MG132 prevented development of thermal sensitivity as well as downregulation of spinal GTs in CFA-injected rats. 

Significantly reduced spinal GT uptake activity was observed on the ipsilateral side after injection with CFA or formalin [44]. Reduction of spinal GTs has been reported to induce accumulation of glutamate in the synaptic cleft and contribute to development and maintenance of inflammatory and neuropathic pain [18, 34]. A novel finding from this work is the observation that decreased expression of GLAST and GLT-1 recovered by EA stimulation in CFA-injected rats and treatment with both EA and proteasome inhibitor could result in upregulation of GLAST and GLT-1 expression. Consequently, EA may result in blockade of activation of spinal astrocytes and downregulation of spinal GTs expression in CFA-induced inflammatory pain, and these may thus enhance glutamate uptake at the synapse and result in an antinociceptive effect. Therefore, further molecular and cellular understanding of spinal GTs underlying EA analgesia may reveal potential therapeutic targets for treatment of pathological pain.
